# Recent advances in membrane technologies applied in oil–water separation

**DOI:** 10.1186/s11671-024-04012-w

**Published:** 2024-04-15

**Authors:** Jialu Huang, Xu Ran, Litao Sun, Hengchang Bi, Xing Wu

**Affiliations:** 1 In Situ Devices Center, School of Integrated Circuits, East China Normal University, Dongchuan Road, Shanghai, 200241 China; 2 SEU−FEI Nano−Pico Center, Key Lab of MEMS of Ministry of Education, Collaborative Innovation Center for Micro/Nano Fabrication, Device and System, Southeast University, Nanjing, 210096 China

**Keywords:** Membrane separation technology, Water purification, Oil water separation, Antifouling, Stimulus responsiveness

## Abstract

Effective treatment of oily wastewater, which is toxic and harmful and causes serious environmental pollution and health risks, has become an important research field. Membrane separation technology has emerged as a key area of investigation in oil–water separation research due to its high separation efficiency, low costs, and user-friendly operation. This review aims to report on the advances in the research of various types of separation membranes around emulsion permeance, separation efficiency, antifouling efficiency, and stimulus responsiveness. Meanwhile, the challenges encountered in oil–water separation membranes are examined, and potential research avenues are identified.

## Introduction

**Fig. 1:**
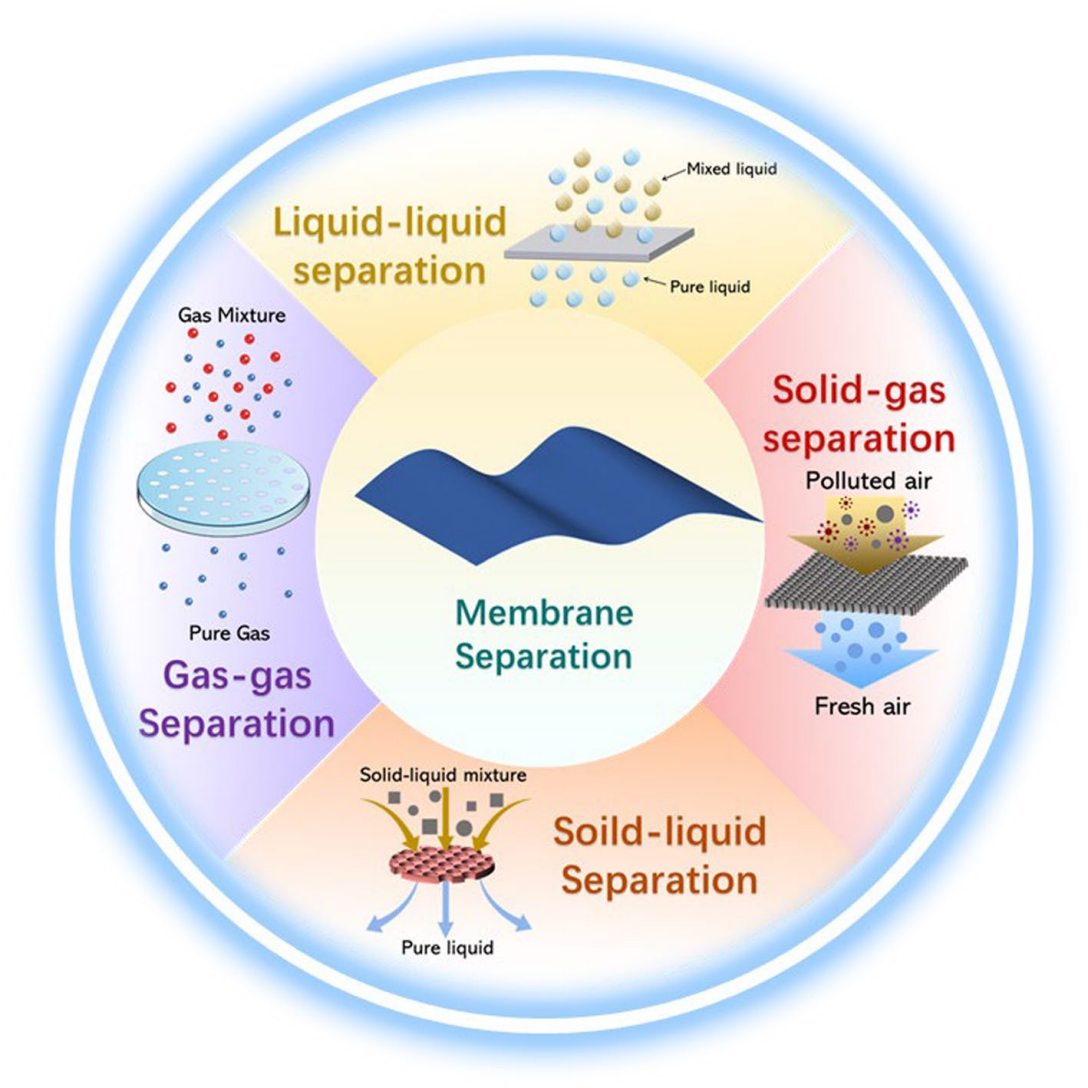
Applications schematics of the membrane separation technologies

Membrane separation technology is a highly efficient method of separation that combines materials science and media separation technology. It offers several advantages, including high separation efficiency, simple equipment, energy savings, room temperature operation, and no pollution [1–4]. The technology is widely used in various industrial fields, particularly in the areas of food, medicine, and biochemicals [5–9]. As shown in Fig. 1, membrane separation technology can be used for gas–gas, solid–gas, solid–liquid, and liquid–liquid separation depending on the substance to be separated [10–13]. Membranes can be classified as either polymer (organic) or inorganic based on their materials. Polymer membranes are commonly used in membrane separation technology due to their high selectivity, ease of control, and uniform structure [14–16]. According to the polymer materials, the polymer membranes can be divided into polyamide (PA) membranes [17], polysulfone (PSU) membranes [18], polyvinylidene fluoride (PVDF) membranes [19], polyethersulfone (PES) membranes [20] and polyacrylonitrile (PAN) membranes [21]. Inorganic membranes have a wide range of applications in the field of membrane separation due to their high temperature stability, chemical inertness, and resistance to contamination [22–24]. Therefore, based on the advantages of organic and inorganic membranes, both have been widely studied in oil–water separation.

Currently, 10% of the global populace in countries face high or serious water scarcity due to population expansion and water pollution, especially oily wastewater from different industries [25]. Recent studies demonstrate the generation of a substantial volume of oily wastewater from oil and gas extraction, processing, and transportation operations. For instance, shale oil extraction yields around 15 billion barrels of oily wastewater in the United States each year [26]. Moreover, failure to appropriately manage contaminated oily wastewater can result in a global economic loss of $4.5 trillion by the year 2050 [27]. Therefore, it is now critically important to separate oil from oily wastewater. Based on the diameter (*d*) of oil in oily wastewater, it can be categorized into free oil (*d* > 150 μm), dispersed oil (20 μm ≤ *d* ≤ 150 μm), and emulsified oil (*d* < 20 μm) [28–30]. For free oil and dispersed oil, traditional water treatment methods, such as gravity separation, centrifugation, flotation, adsorption, and coagulation [31–36], can easily separate them from the oil–water mixture. However, common standard separation technologies find emulsified oil challenging to remove because surfactants stabilize the minuscule oil droplets, significantly lowering the interfacial tension between oil and water [28, 37–39]. Consequently, it is evident that the development of membrane separation technology is essential to treat oil–water emulsion.

Oil–water separation is a common liquid–liquid separation technique. Compared to traditional methods, Membrane separation technology allows for precise separation by adjusting the pore size according to requirements. The process is driven by pressure differences, making it simple to operate and energy-efficient [40, 41]. Furthermore, the membranes in membrane separation technology are carefully composed of suitable materials to treat oily wastewater effectively [42–44]. The ideal separation membranes usually satisfy the following performance requirements: high permeance, high separation efficiency, high antifouling efficiency. The researchers have conducted extensive research on improving each performance [38, 45–49]. However, the membranes with the aforementioned excellent performances simultaneously are still hard to achieve due to a lack of comprehensive understanding of each performance. When assessing pressure-driven separation processes, key factors like pH, transmembrane pressure (TMP), pore characteristics, fouling, temperature, and feed composition are critical [50–55]. PH affects membrane stability, while TMP impacts permeation rates and selectivity. Pore size influences permeability and selectivity, and fouling reduces membrane flux and selectivity. Temperature affects fluid properties and membrane performance, and feed composition directly influences separation efficiency. Understanding and optimising these factors can lead to improved performance and efficiency in separation processes. Therefore, it is necessary to provide a detailed description of each performance and its underlying mechanisms. Several existing reviews on oil–water separation membranes are mainly elaborated from the perspectives of materials and applications [42, 56]. However, no review currently comprehensively and systematically introduces the performances of emulsion permeance, separation efficiency, antifouling efficiency, and stimulus responsiveness.

**Fig. 2:**
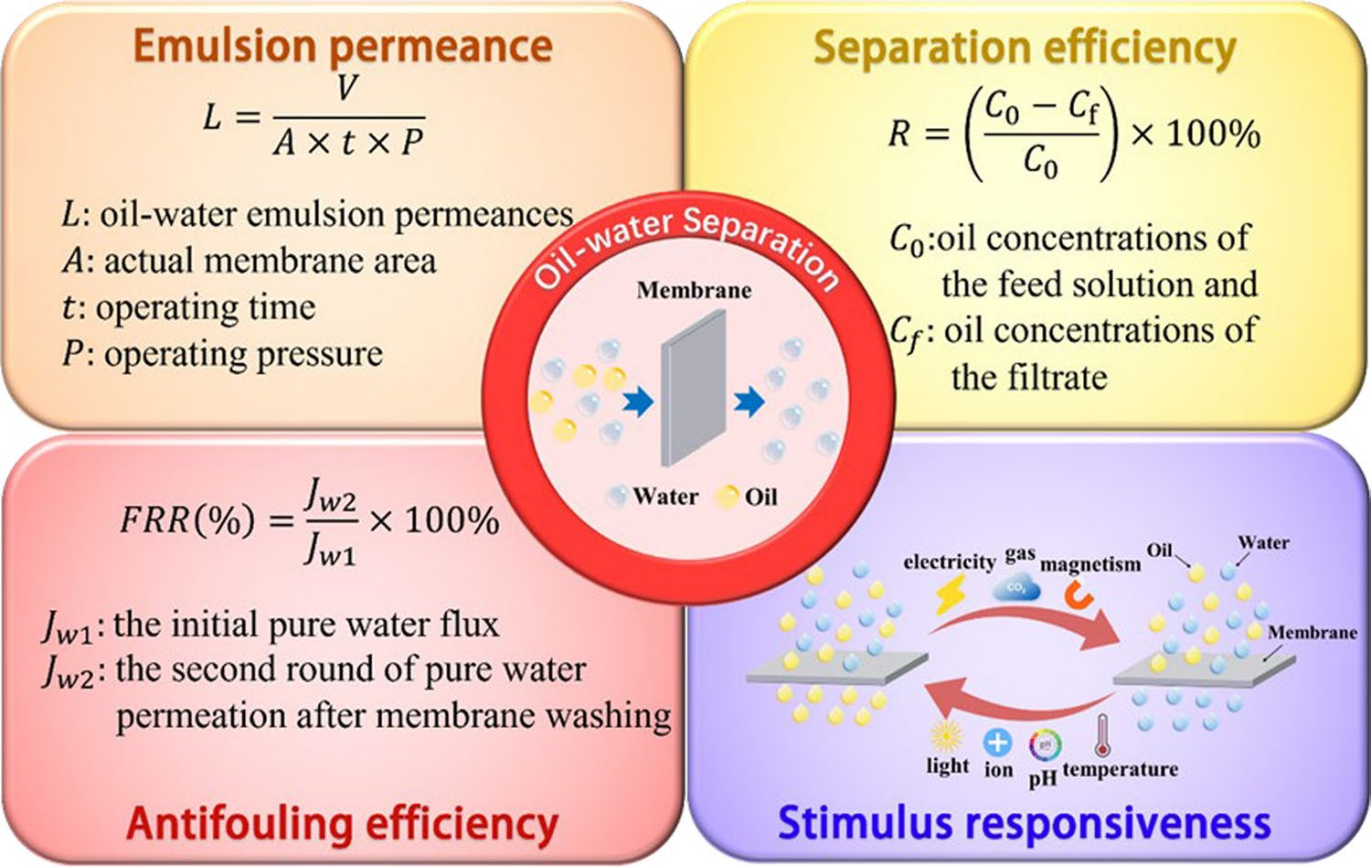
Properties of oil–water separation membranes

This review summarizes the latest research on oil–water separation membranes with diverse properties to emulsion permeability, separation efficiency, fouling resistance, and stimulus responsiveness (Fig. 2). These properties are essential for evaluating the separation performance of membranes. The detailed definition of these properties is described in Fig. 2. Each property is elaborated based on typical works from preparation methods, morphological characteristics, structural features, and work mechanisms of the membranes. Moreover, the issues encountered by oil–water separation membranes are highlighted, and the paper proposes future directions for development.

## Emulsion permeance

The emulsion permeance of oil–water separation membranes directly reflects the separation process's efficacy. It is crucial to enhance the membrane's emulsion permeance to manage the large volume of oily wastewater. Additionally, increased emulsion permeance results in higher water flow through the membrane per unit of time. This decreases the water residence time on the membrane surface, reduces its resistance to flow, and decreases the pressure difference between the two sides of the membrane. As a result, the operating pressure is reduced, lowering energy consumption and operating costs [57–60]. Thus, improving the emulsion permeance of membranes has become a crucial aspect in advancing oil–water separation membranes.

Permeation theory suggests that the water permeation rate through a membrane is directly linked to the membrane's porosity and inversely proportional to its thickness [61]. Optimizing these physical properties is crucial in designing effective membranes for oil–water separation. The perfect membranes have high flux while maintaining good selectivity. It has been found that researchers have made progress towards achieving this goal. Electrostatic spinning is an effective technology for constructing membranes with high permeation. The electrostatic spinning parameters can be fine-tuned to precisely adjust the fibers'''' diameter in the resulting fiber membrane, effectively separating the oil–water mixture. Nanofibrous membranes produced through electrospinning technology exhibit a thin separation layer with nanoscale thickness and high porosity (> 90%), which endows the membrane with excellent emulsion permeability during the process of oil–water separation [62–65].

**Fig. 3:**
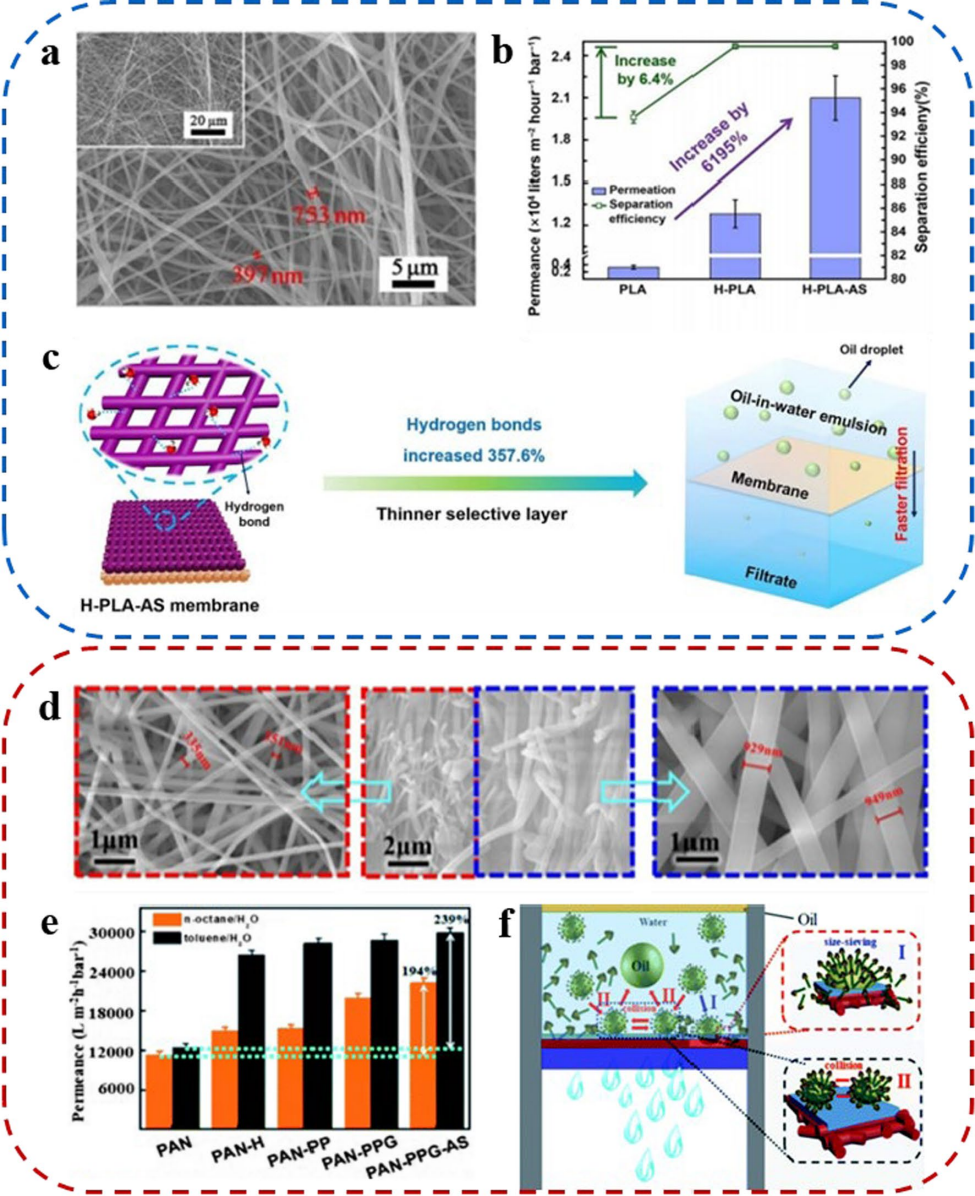
**a** FE-SEM of the supporting layer of the H-PLA-AS membrane (14 wt% of PLA–10 wt% of PEO). **b** Separation performance of PLA-based membranes for n–octane–in–water emulsions. **c** The possible mechanism of the improvement in permeance of H-PLA-AS membranes. **d** SEM micrographs of PAN-PPG-AS. **e** Emulsion permeances of all kinds of PAN-based membranes for n-octane-in-H_2_O emulsion (N/E) and toluenein-H_2_O emulsion (T/E). **f** Schematic diagram of the oil–water separation process

By utilizing electrospinning technology, *Shao* and his colleagues developed biodegradable supersaturated membranes composed of polylactic acid nanofibers and polyethylene oxide hydrogel (H-PLA-AS membranes) [66]. The membrane exhibited a uniform stacking structure of nanofibers with micron-sized bead formations as observed through field emission scanning electron microscopy (FE-SEM) (Fig. 3a). Figure 3b illustrates a noteworthy increase in both emulsion permeability and separation efficiency of H-PLA-AS membranes when compared to the original PLA membranes. Specifically, the emulsion permeability of H-PLA-AS membrane increased 61.9 times (2.1 × 10^4^ L m^−2^ h^−1^ bar^−1^), and the separation efficiency achieved an impressive 99.6%. Figure 3c shows the potential Mechanism for the improved permeation performance of the H-PLA-AS membrane. Specifically, PEO increased the number of hydrogen bonds, enhancing the hydrophilicity and Permeability to water. Furthermore, the thinner selective layer also contributed significantly to the heightened Permeability.

*Cheng* and his co-workers designed a super hydrophilic PAN asymmetric nanofibrous membrane (PAN-PPG-AS) by electrostatic spinning with an in situ hybridized multi-hydrophilic functional network as the sole selective layer [67]. Microscopic examination revealed that the coarse nanofibers of the support layer were situated beneath the fine nanofibers of the selective layer in the PAN-PPG-AS membrane's cross-section. The diameter of the nanofibers in the support layer measured approximately 900 ± 20 nm, and in the selective layer was about 340 ± 20 nm (Fig. 3d). The PAN-PPG-AS membranes exhibited higher permeation fluxes for n-octane-water emulsion and toluene-water emulsion, achieving 22,206 L m^−2^ h^−1^ bar^−1^ and 29,840 L m^−2^ h^−1^ bar^−1^, respectively. These results were 239% and 194% higher than the pure PAN membranes (Fig. 3e). Furthermore, the authors have illustrated that the separation of oil–water emulsions is influenced not only by the size-sieving effect but also by the disparity in inherent hydrophilicity between the membrane and the liquid (Fig. 3f).

## Separation efficiency

Separation efficiency is another crucial metric when assessing the efficacy of oil–water separation membranes. Oil–water separation membranes have displayed remarkable separation efficiency for free and dispersed oil–water mixtures, surpassing 95% [68–70]. However, their separation performance for emulsified oil–water mixtures with smaller droplet sizes of the dispersed phase is not good, especially for stable emulsions with droplet sizes less than 20 μm, which are stabilized by surfactants, and this requires a smaller pore size and strong wettability for the oil–water separation membranes [71–73].

**Fig. 4:**
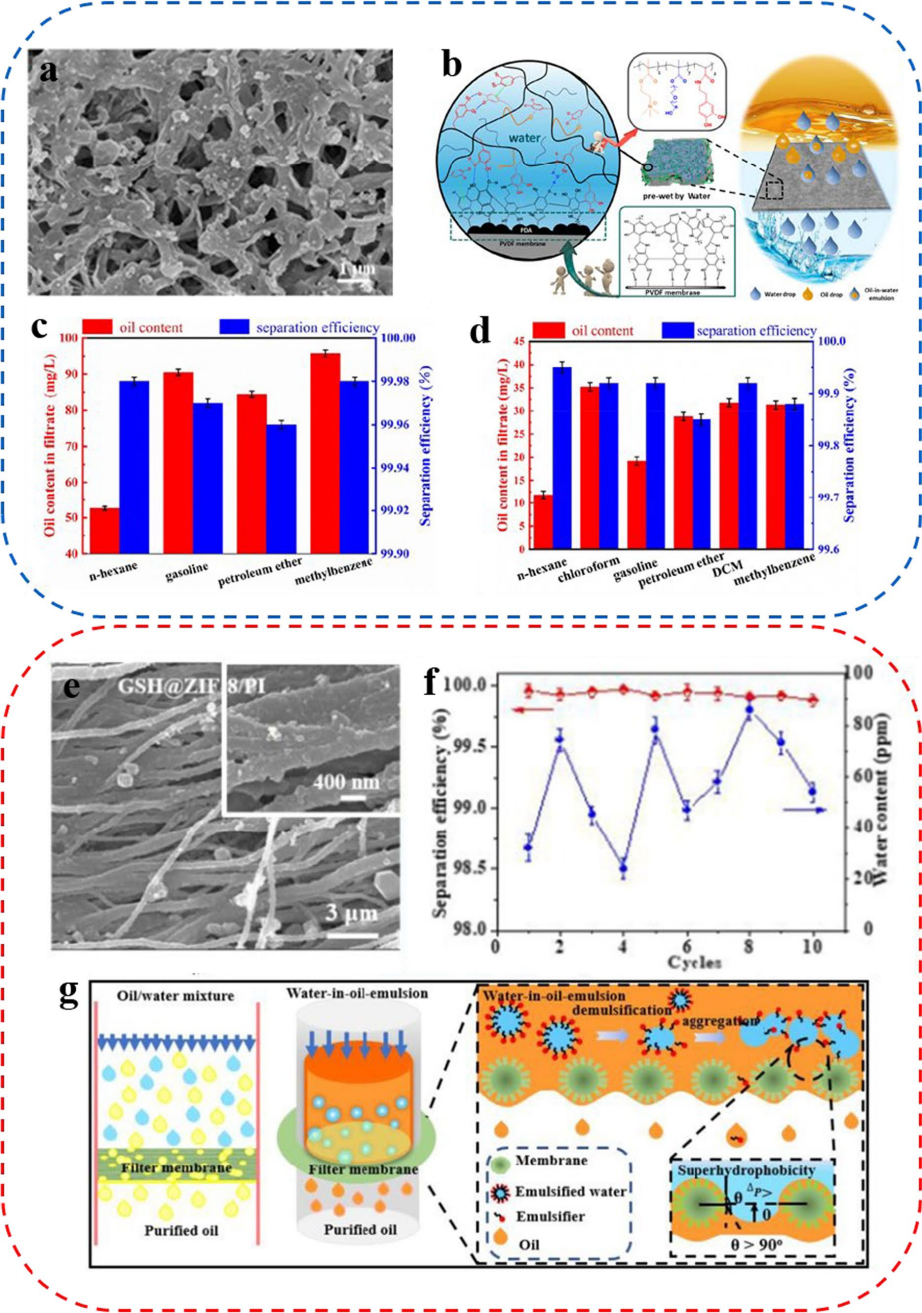
**a** SEM micrograph of the CFHP/PDA-coated membrane. **b** Working mechanism of the CFHP/PDA-coated membrane in oil–water separation. **c** Separation efficiency towards different oil–water mixtures. **d** Separation efficiency of different oil-in-water emulsions. **e** SEM image of ZIF-8@GSH/PI nanofibrous membrane. **f** The cycle separation efficiency of the membrane for various water-in-oil emulsions. **g** Schematic diagram for the separation of oil–water mixture and water-in-oil emulsion

More and more researchers are preparing polymer membranes with high separation efficiency by various methods. For example, some researchers have prepared ultrafiltration and nanofiltration membranes by using green solvents through phase inversion methods [74–76]. In addition, Xu et al. [77] proposed a practical method based on mussel-inspired dip-coating for building a stable hydrophilic polymer network on membrane surfaces, which involved sequential immersion of the substrate membrane into aqueous solutions of polydopamine (PDA) and catechol-functionalized hydrophilic polymer (CFHP). SEM revealed the formation of rough hierarchical nanostructures on the surface of the prepared CFHP/PDA-modified membranes (Fig. 4a). After being pre-wetted with water, the polymer network swells with water to create a thin and stable aqueous film layer, serving as a hurdle to oil penetration (Fig. 4b). Figure 4c and d demonstrate that the CFHP/PDA modified membranes separated various oil–water mixtures and oil-in-water emulsions stabilized by surfactants effectively with outstanding separation performance (99.98% separation efficiency).

*Fu* and his co-workers created a new polyimide (PI) nanofiber membrane to efficiently purify oily wastewater [78]. The membrane incorporated a composite of zeolitic imidazolate framework-8@thiolated graphene (ZIF-8@GSH), which was formed using a straightforward process of electrostatic spinning and in situ hydrothermal synthesis. The microscopic characterization revealed that the PI nanofiber surface gradually became covered by a continuous ZIF-8@GSH composite as ZIF-8 develops in situ (Fig. 4e). As demonstrated in Fig. 4f, the ZIF-8@GSH/PI exhibited outstanding separation efficiency (> 99%), low water content (< 100 ppm), and durability. Figure 4g depicts the schematic membrane diagram implemented for separating oil–water mixtures and water-in-oil emulsions. When separating oil–water mixtures, the superhydrophobicity of the membrane enables water droplets to repel easily from its surface. In contrast, oil droplets progressively wet the membrane and infiltrate the micron/nanoscale grooved portion constituted by the membrane's super-hydrophilicity. The successful separation of oil and water mixtures has been achieved. However, when dealing with surfactant-stabilized water-in-oil emulsions, the membrane's superhydrophobicity and superlipophilicity cause the emulsion to become destabilized upon contact with the membrane surface. On the other hand, surfactant prevents water droplet aggregation and allows the membrane to effectively block the micro/nano water droplets enclosed in the oil. Consequently, the capillary effect efficiently captures and spreads out the oil.

Membrane distillation (MD) shows potential for water treatment, particularly in seawater desalination, as it can separate water from contaminants through vapor-phase transport using hydrophobic membranes [79, 80]. MD operates at low temperatures, preserving water quality and energy efficiency, and exhibits high selectivity, making it suitable for various applications [81, 82]. The modular and scalable nature of MD allows for deployment in diverse settings, from decentralized systems to large-scale industrial use [83]. However, MD faces challenges such as lower water flux and fouling, which limit its throughput and increase operational costs [84, 85]. To address these limitations, future trends in incorporating inorganic materials focus on enhancing membrane resistance to wetting and fouling. Strategies to improve mechanical strength, selectivity, and resistance to fouling and scaling include integrating superhydrophobic coatings, nanomaterials, and nanocomposite membranes that utilize graphene oxide or metal–organic frameworks (MOFs) [86, 87].

Furthermore, researchers have developed membranes with high separation efficiency for treating water-in-oil emulsions. For instance, Liu et al. created a new tubular polyvinyl chloride (PVC) hybrid nanofiber membrane with a three-dimensional structure composed of three-dimensional microspheres and two-dimensional nanofibers interwoven by an electrostatic spinning process [88]. The membrane that has been prepared exhibits a high separation efficiency of over 95% and excellent reusability in water-in-oil emulsions. Huan and his colleagues successfully created composite membranes with a hierarchical structure using electrostatic spinning technology [89]. These membranes consist of a selective layer of polyvinylidene difluoride (PVDF) nanofibers, a layer of polymethylmethacrylate (PMMA) microspheres, and a support layer of polyacrylonitrile (PAN) nanofibers. The membrane exhibits high separation efficiency, porosity, and flux, and can effectively separate water-in-oil emulsions through gravitational means.

Improving the balance between selectivity and permeability is a crucial objective in the development of separation membrane materials. To enhance selectivity without compromising permeability, several strategies can be employed. One such strategy is the chemical crosslinking of polymer chains, which can improve membrane stability and selectivity [90]. Crosslinking polymers usually reduces the mobility of the chains, thereby limiting the diffusion of larger molecules through the membrane matrix. Functionalization of polymeric membranes can increase their affinity for specific molecules, thereby improving selectivity, which can be achieved by introducing specific functional groups [91]. Mixed matrix membranes (MMMs) can be fabricated by incorporating inorganic fillers into a polymer matrix. The addition of filler molecules provides additional diffusion paths, improving selectivity while maintaining high permeability [92]. Thin film composite (TFC) membranes can achieve high selectivity without sacrificing permeability by optimizing the thickness and composition of the selective layer [93]. Advanced processing techniques such as phase inversion, electrospinning and layer-by-layer deposition allow precise control of the structure and morphology of the membranes, improving selectivity by reducing defects and enhancing the molecular sieve effect [94–96]. Researchers are using these strategies to overcome the inherent limitations of the trade-off between selectivity and permeability.

## Antifouling efficiency

Membrane fouling is a common problem in water purification [97, 98]. Membrane fouling occurs when oil droplets adhere to the membrane surface or gather in the pore channels, preventing water from passing through the membrane, thereby reducing the water permeation flux, decreasing the efficiency of the separation process, and increasing energy consumption [38, 99, 100]. Membrane fouling is a self-accelerating process that degrades membrane performance and prevents stable long-term operation [74, 101]. Frequent chemical cleaning or air flushing is necessary to solve this issue [102–104]. However, these approaches result in a noteworthy cost increase and a reduction in the membrane's service life. Therefore, producing oil–water separation membranes with proficient fouling resistance has emerged as a popular research subject.

**Fig. 5:**
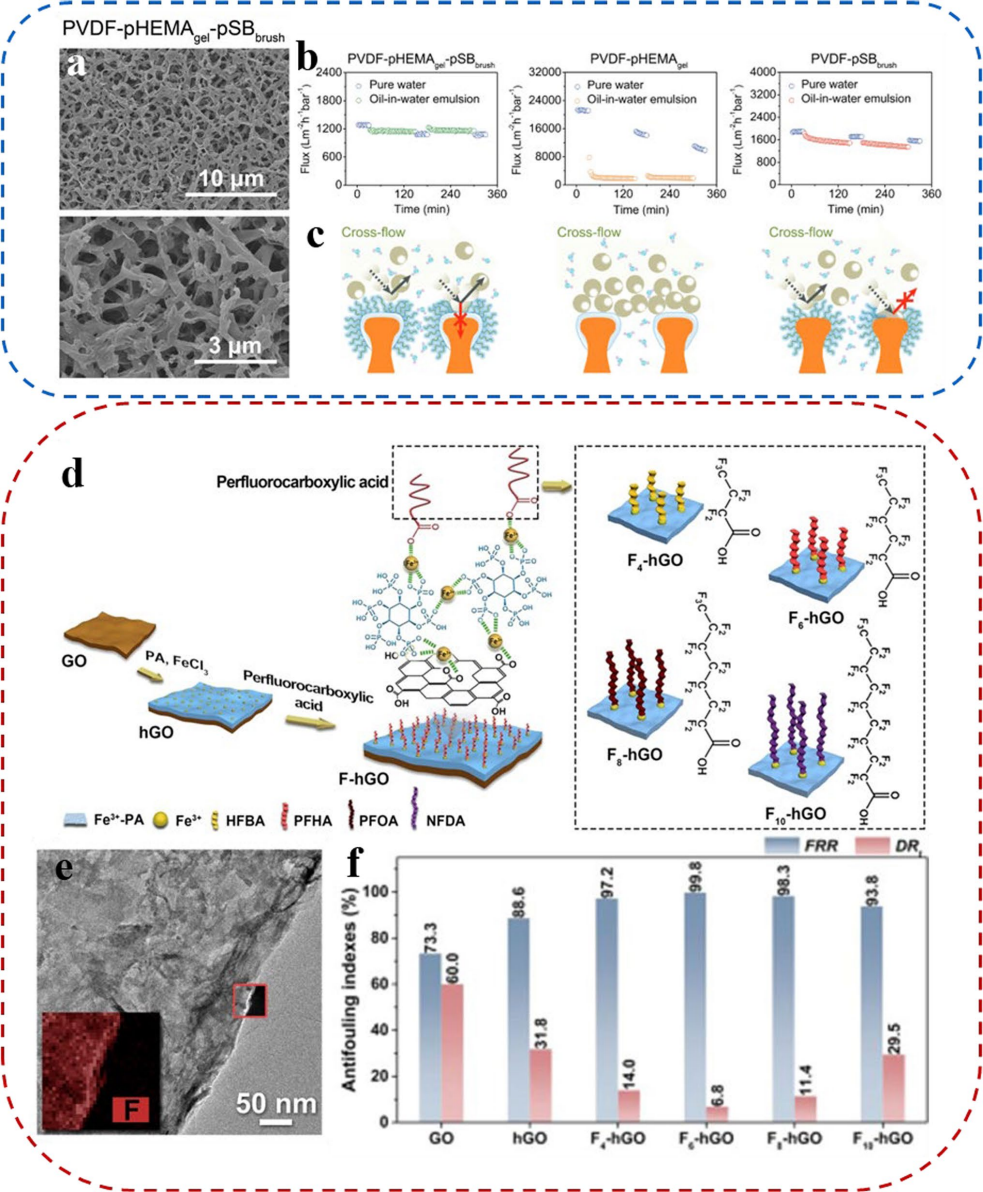
**a** SEM images of PVDF-pHEMA_gel_-pSB_brush_. **b** Real-time water permeating flux variation of PVDF-pHEMA_gel_-pSB_brush_, PVDF-pHEMA_gel_ and PVDF-pSB_brush_, respectively. **c** Schematic illustrating "double-defense" Mechanism against oil fouling of the PVDF-pHEMA_gel_-pSB_brush_ membrane. **d** Schematic diagram of the production of the F-hGO membranes. **e** TEM and corresponding EDS mapping images of the F_6_-hGO membrane without the substrate. **f** Antifouling performance of the GO, hGO, and F-hGO membranes

*Dong* and his colleagues propose a "double-defense" design, where poly amphiphilic brushes and hydrogels are layered on the membrane surface to form an effective oil barrier [105]. The PVDF-pHEMA_gel_-pSB_brush_ membrane prepared by this method had outstanding resistance to oil contamination and self-cleaning capability. Figure 5a depicts the membrane microstructure as observed via scanning electron microscopy. The PVDF-pHEMA_gel_-pSB_brush_ membrane contained micron-scale sponges with a thin polymer coating on the ridges surrounding the holes. PVDF-pHEMA_gel_-pSB_brush_, PVDF-pHEMA_gel,_ and PVDF-pSB_brush_ membranes were evaluated for their separation efficacy of surfactant-stabilized oil-in-water emulsions under staggered flow conditions (Fig. 5b). The test results indicated strong stability in the separation performance of the PVDF-pHEMA_gel_-pSB_brush_ membrane for two hours under an applied pressure of 0.2 bar. Permeate flux remained consistently above 1100 L m^−2^ h^−1^ bar^−1^. The membrane boasted a high effective flux recovery rate (FRR) of 99.1% after two filtrations, demonstrating excellent antifouling capabilities. Figure 5c illustrates the "double defense" mechanism of the PVDF-pHEMA_gel_-pSB_brush_ membrane. In this membrane, the outermost flexible poly(sulfobetaine) (pSB) brushes were firmly hydrated to prevent oil adhesion and formed the "first defense" layer. Another "second defense" layer is provided by the poly(hydroxyethyl methacrylate) hydrogel overlay film (PVDF-pHEMA_gel_) to enhance resistance against the oil. A "double defense" barrier is effectively established on the surface of the membrane, which highly covers and repels oil adhesion and accumulation.

The solution to the oil–water membrane fouling issue is to minimize the interfacial interaction between the membrane surface and the pollutants [106–108]. The prevalent method utilizes hydrophobic substances, typically fluorine-based and silane materials, applied onto hydrophilic surfaces, creating an appropriate amphiphilic interface that promotes fouling resistance [109–112]. Jiang et al. proposed a molecular engineering approach involving hydrophobic chains [113]. They sequentially assembled hydrophilic phytic acid (PA) and hydrophobic perfluorocarboxylic acids on the graphene oxide (GO) surface, forming a surface having both continuous hydrophilic and discontinuous hydrophobic regions (F-hGO membrane). By adjusting hydrophobic chain length, interfacial interactions between the membrane and oil droplets were regulated, leading to improved antifouling performance of the membrane (Fig. 5d). Figure 5e demonstrates that perfluorocarboxylic acid is uniformly distributed across the membrane surface as observed through energy dispersive X-ray spectroscopy (EDS). The prepared membranes were evaluated for fouling resistance by separating the hexadecane-in-water emulsion. As shown in Fig. 5f, the F-hGO membrane demonstrated FRR up to 99.8% and the total flux decline ratio (DR_t_) down to 6.8%. These results reflected a considerable improvement over GO membranes and phytate-modified membranes (hGO), with a 1.4-fold and 1.1-fold increase in FRR and a 90% and 80% reduction in DR_t_, respectively. The data indicates that incorporating perfluorocarboxylic acid can improve GO membranes' fouling resistance.

In addition, an increasing number of researchers are creating oil–water separation membranes with high fouling resistance through surface modification of ceramic membranes. Ceramic membranes are an inorganic material that can be used for oil–water separation in harsh environments due to their high chemical stability, excellent mechanical strength, and super hydrophilicity. The performance of ceramic membranes can be effectively improved and oil–water separation membranes with high fouling resistance can be prepared by modifying their surface. Gao and Xu successfully constructed nanostructured silver coatings grafted with hexadecanethiol on the surface of ceramic membranes using a dopamine-assisted nanoparticle encapsulation process [114]. The modified membranes exhibited superior anti-fouling properties compared to the original membranes. Fan et al. developed anti-fouling ceramic membranes using a two-step grafting method to attach amphoteric ions to the surface of the ceramic membranes [115]. This modification resulted in a significant improvement in antifouling performance by reducing irreversible contamination during oil–water emulsion separation.

Furthermore, incorporating nanofillers into membranes is also a promising approach to improve the antifouling efficiency and separation performance. Nanofillers, such as nanoparticles or nanotubes, can enhance membrane properties through various mechanisms, including increased surface area, improved mechanical strength, and enhanced selectivity [116]. Jose R. Aguilar Cosme et al. [117] found that inorganic nanoparticles' high adsorption capacity enabled nanocomposite membranes to outperform previous pristine membranes in removing dyes, metal ions, humic substances, and more. In addition, Vantanpour et al. [118] modified cellulose acetate nanofiltration membranes with zeolite imidazoline framework-8 (ZIF-8) nanoparticles for water treatment applications. The study found that the ZIF-8 nanoparticles' high surface area and adsorption capacity reduced fouling caused by organic compounds, thereby enhancing the membrane's stability and fouling resistance.

## Stimulus responsiveness

Stimulus responsiveness plays a crucial role in developing oil–water separation membranes toward more advanced areas. Compared to traditional oil–water separation membranes, which feature fixed pore structure and surface properties, stimuli-responsive oil–water separation membranes can detect, analyze and adapt to various environmental stimuli, resulting in changes in physiochemistry, morphology, structure, and molecular conformation of membranes, which in turn alter the wetting properties of the membrane surface and the liquid transport channels [119–123]. As a result, these membranes can selectively exhibit either hydrophobicity or hydrophilicity, thereby achieving controlled oil–water separation. Standard external stimuli, such as electricity [124, 125], gas [126, 127], ion [128], light [129, 130], magnetism [131], pH [132, 133], and temperature [134], each possess unique response mechanisms and could potentially be used in developing stimuli-responsive membranes for separating oil and water.

### Gas stimuli-responsive membranes

Gas stimuli-responsive membranes can demonstrate switchable wettability in response to external gas stimuli. These membranes are cost-effective, environmentally friendly, easily reversible, and uncontaminated solutions [135, 136]. Gases commonly used as stimuli include oxygen (O_2_), carbon dioxide (CO_2_), nitrogen (N_2_), and methane (CH_4_). CO_2_ is regarded as the most desirable gas stimulant among these irritants because of its non-toxicity, low cost, and renewability advantages [137–139].

**Fig. 6:**
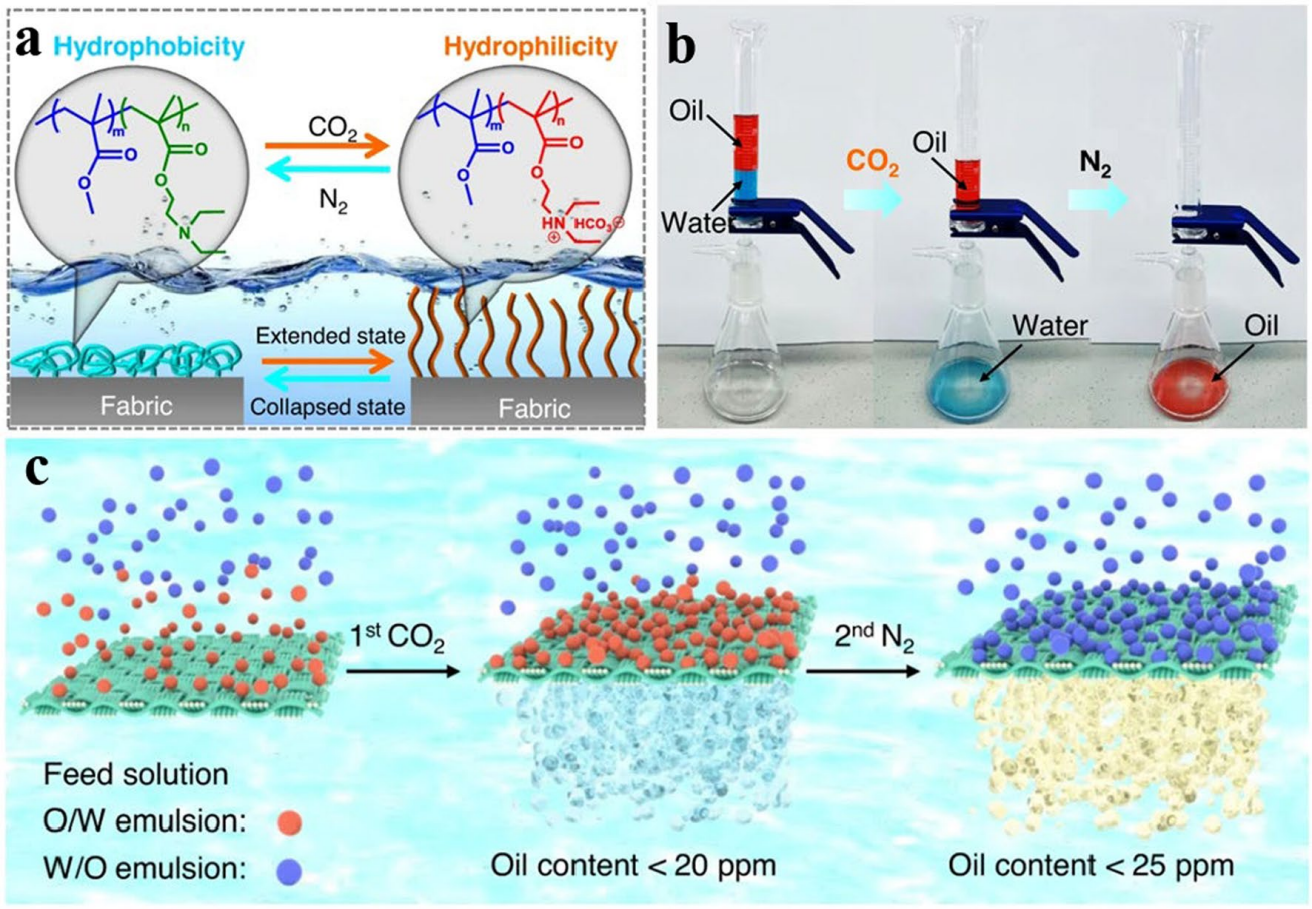
Is a schematic illustration of the surface-wetting mechanism mechanism of the membrane under CO_2_/N_2_ stimulation. Separation process of the immiscible oil–water combination under CO_2_/N_2_ stimulation at 25 °C. **c** Multi-emulsion separation process under CO_2_/N_2_ stimulation.

Drawing inspiration from the natural capillary phenomenon, Dong et al. developed a strategy to fabricate CO_2_-sensitive membranes that can effectively separate different oil and water systems, driven by capillary forces and involving a limited self-assembly process that results in scalable and robust membranes [140]. The prepared membrane attached the CO_2_-responsive copolymer poly(diethylaminoethyl methacrylate-co-methyl methacrylate (PMMA-*co*-PDEAEMA) to the basement membrane's surface uniformly using capillary force. Further, the wettability of the membrane was altered by protonating and deprotonating amine groups in the PDEAEMA chain segments of the copolymer under the stimulation of CO_2_ or N_2_ (Fig. 6a). As shown in Fig. 6b and c, the produced membrane can effectively separate various oil–water systems, such as immiscible mixtures, oil-in-water emulsions, water-in-oil emulsions, with exceptional separation efficiency (> 99.9%), recoverability, and self-cleaning properties.

### pH stimuli-responsive membranes

pH stimuli-responsive membranes have several benefits, such as eco-friendliness, low energy consumption, outstanding reversibility, and fast response, rendering them increasingly prevalent in molecular recognition, biosensors, and material separation fields [141–145]. The pH-stimulated response behavior of membranes relies on pH-sensitive polymers or copolymers applied to the membrane surface. Common polymers or copolymers used in pH stimuli-responsive membranes include poly(acrylic acid) (PAA), poly(methacrylic acid) (PMAA), poly(dimethylaminoethyl methacrylate) (PDMAEMA), and so on. These polymers or copolymers acquire or release protons depending on the pH conditions, which alters the wetting properties of the membrane surface and enables the membrane to switch between hydrophilic and hydrophobic states [146–150].

**Fig. 7:**
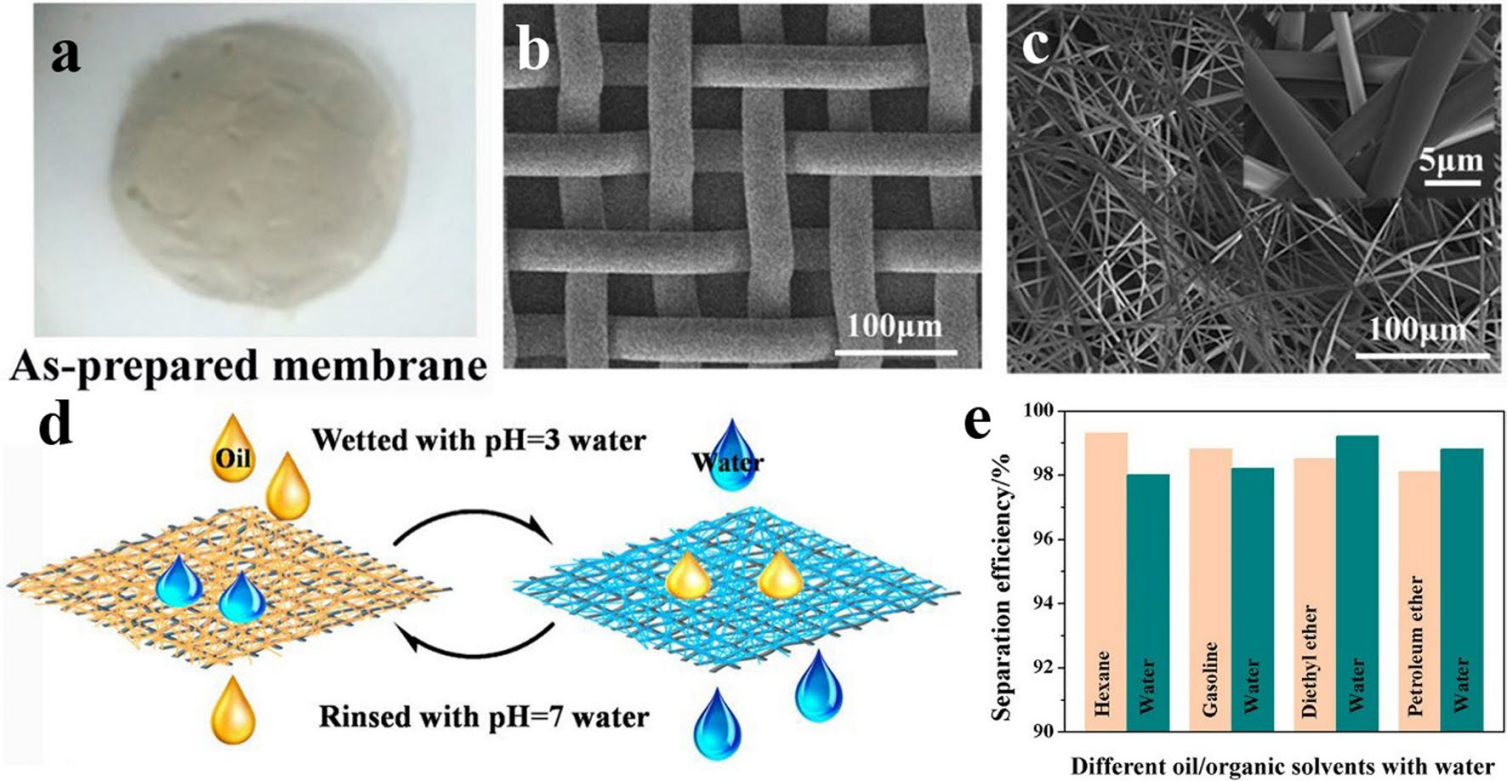
**a** The picture of the as-prepared PMMA-*b*-P4VP fiber membrane. **b** SEM image of original stainless steel mesh. **c** SEM images of fiber-coated stainless steel mesh. **d** Schematic illustration of pH switchable oil–water separation. **e** Separation efficiency of different mixtures

*Luo* and his co-workers generated membranes that respond to PH stimuli via precipitation of the pH-responsive copolymer poly(methyl methacrylate)-block-poly(4-vinylpyridine) (PMMA-*b*-P4VP) onto stainless steel mesh using electrostatic spinning (Fig. 7a) [151]. As illustrated in Fig. 7b and c, the electrostatic spinning led to integrating a high-density, fiber-based layer onto the surface of the stainless steel mesh, forming the three-dimensional macroporous lattice structure that enhances the liquid transport rate within the membrane. Figure 7d illustrates the oil–water separation process of the membrane in response to pH stimulation. When the membrane was wetted with acidic water (PH = 3), the pyridine group of P4VP was protonated and gradually extended to the membrane surface. As a result, the membrane transitioned from being initially hydrophobic-oleophilic to hydrophilic-oleophobic. Furthermore, the membrane retained superior separation efficiency in both hydrophobic and hydrophilic conditions (Fig. 7e).

### Temperature stimuli-responsive membranes

Temperature stimuli-responsive membranes are created by modifying membranes with temperature-responsive polymers that correspond to fluctuations in external temperature, resulting in transformed surface or internal structures of the membrane [152–154]. These membranes possess a critical solution temperature (CST), which can be classified into lower and upper critical solution temperatures (LCST, UCST). Temperatures below or above the CST will prompt the thermo-responsive polymer chains on the surface of the membrane to either inflate or deflate in solution, subsequently influencing the porosity of the membrane and thereby modifying water flux, resulting in changes in the membrane's hydrophilicity and hydrophobicity [145, 155–158].

**Fig. 8:**
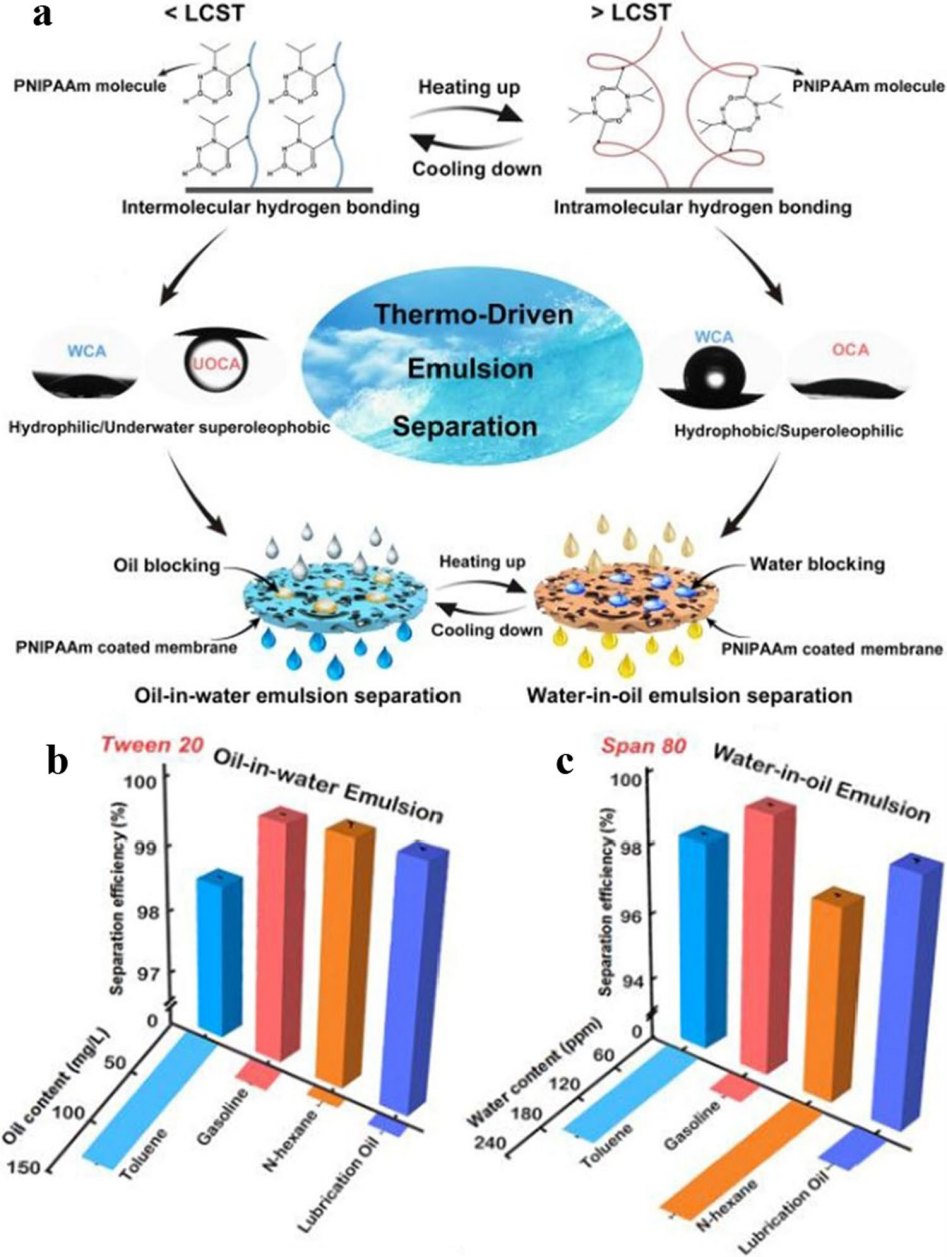
**a** The manufacture of the PNIPAAm coated membrane and the Mechanism of the thermo-responsive membrane. **b** Separation efficiency of the membrane for oil-in-water emulsions. **c** Separation efficiency of the membrane for water-in-oil emulsions

Zhang et al. [159] used a hydrothermal method to create temperature-sensitive poly(N-isopropyl acrylamide)-coated nylon membranes (PNIPAAm coated membranes). As shown in Fig. 8a, the membrane showed hydrophilicity, and the separation of oil-in-water emulsion could be realized when the temperature was lower than LCST. Conversely, when the temperature exceeded the LCST, the membrane became hydrophobic and can separate water-in-oil emulsions. The prepared membranes demonstrated outstanding separation performance for oil-in-water and water-in-water emulsions as shown in Fig. 8b and c.

### Multi-stimuli-responsive membranes

Single-stimulus-responsive membranes react exclusively to a single environmental stimulus signal, while dual- or multi-stimulus-responsive membranes possess the capability to respond to multiple environmental stimuli, thus endowing them with new functions and applications and enabling them to adapt to more complex surroundings [160–163].

**Fig. 9:**
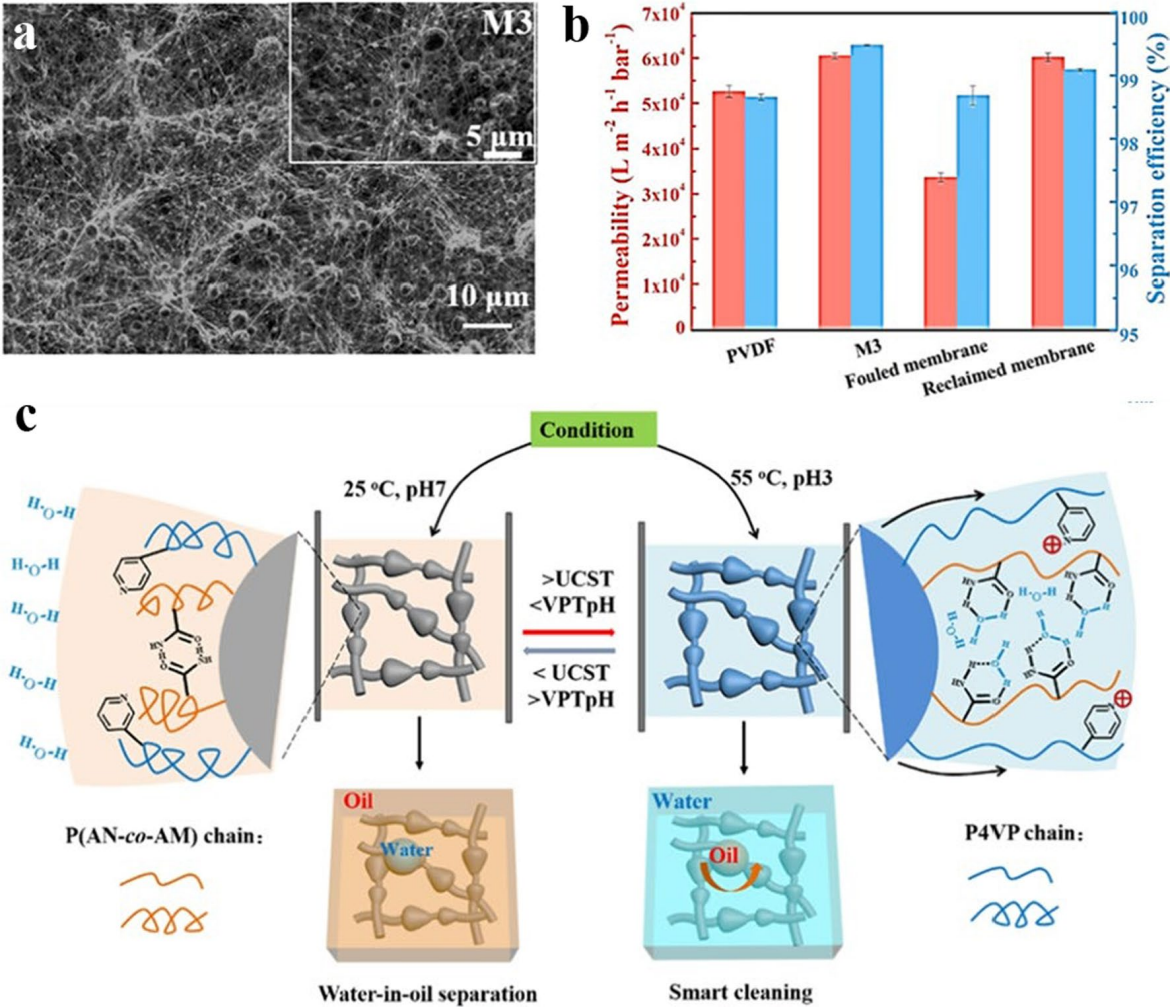
**a** SEM image of the nanofibrous membrane. **b** Permeability and separation efficiency of different membranes. **c** Mechanism of the membrane with the dual UCST-type thermo/pH stimulus responses

Wu and her co-workers developed pH-responsive and UCST-type temperature-responsive nanofiber membranes through a one-step co-mingled electrostatic spinning strategy [164]. SEM examination results showed that the prepared membranes were composed of nanofibers that exhibited a disordered stacked structure (Fig. 9a). Furthermore, micro- and nanospheres were observed on the membrane surface, with connecting points between the microspheres and nanofibers. Figure 9b demonstrates that the nanofiber membrane demonstrated outstanding separation efficacy for oil-in-water emulsions, with a permeate flux of up to 60,528.76 L m^−2^ h^−1^ bar^−1^, and a 99.5% separation efficiency. Furthermore, Fig. 9c depicts that the multi-stimulation response of the membrane is attributed mainly to the temperature responsiveness of poly(acrylonitrile-*co*-acrylamide) (P(AN-*co*-AM)) and the pH response of P4VP. At the temperature of 25 °C (< UCST) and the pH of 7 (> VPTpH), the P(AN-*co*-AM) chains undergo collapse, and the P4VP chains disintegrate due to the deprotonation of N atoms, making the membrane hydrophobic. On the other hand, when the temperature rises to 55 °C (> UCST) and the pH drops to 3 (< VPTpH), the P(AM-*co*-AM) chains gradually dissolve and are encompassed by a multitude of water molecules. The P4VP chains simultaneously increase in size due to the pyridine group protonation. Both of these changes make the membrane become hydrophilic.

### Other stimuli-responsive membranes

**Fig. 10:**
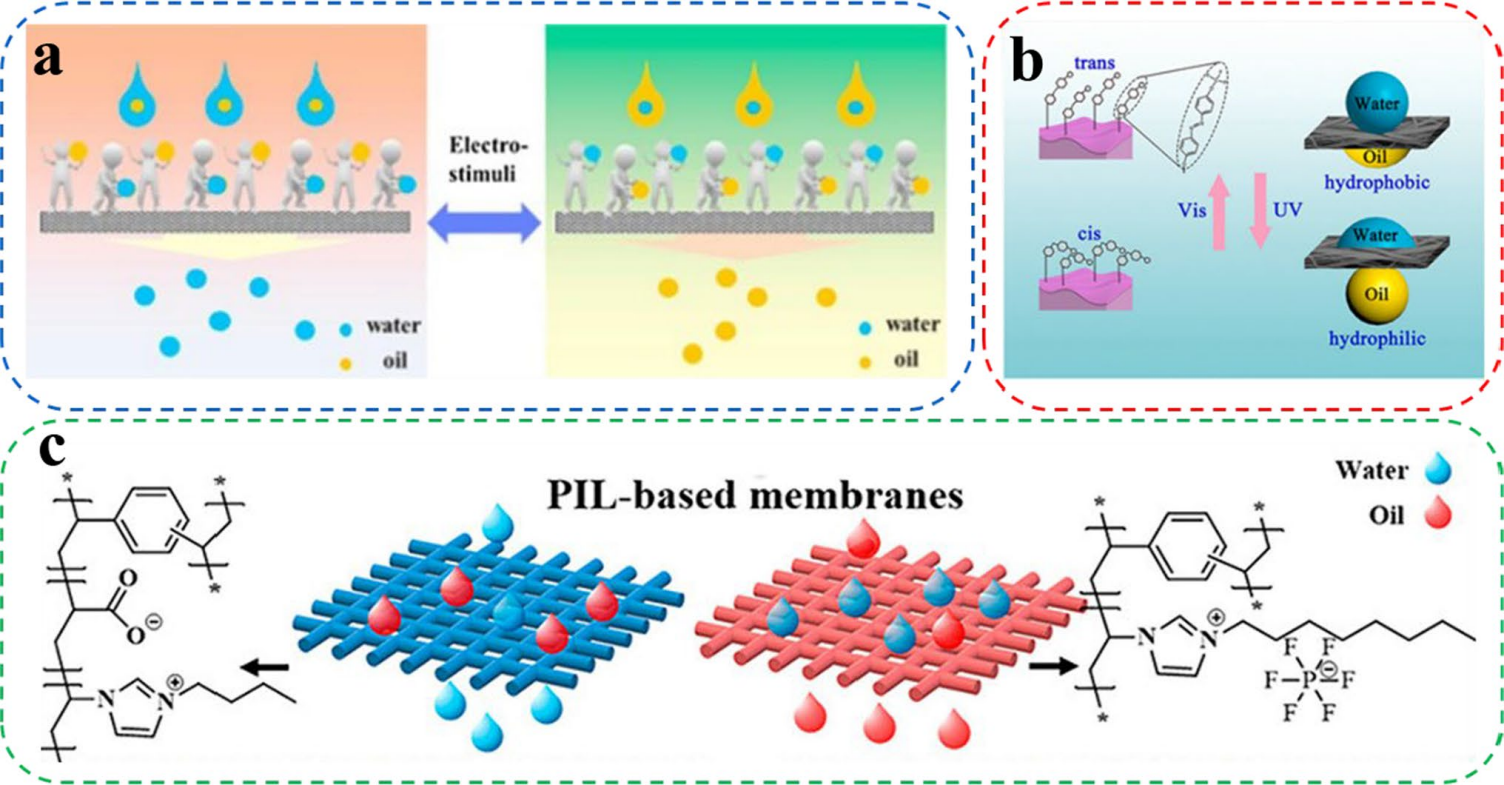
**a** Schematic illustration of the mechanism of the electro-responsive carbon membrane. **b** Schematic illustration of light switchable oil–water separation. **c** Mechanism of the s PIL-based membranes (PILMs)

There are electric stimuli-responsive membranes, light stimuli-responsive membranes, ion stimuli-responsive membranes, and so on. Electric stimuli-responsive membranes comprise electroactive polymeric materials, with precise control over surface wettability achieved by adjusting the contact angle of droplets via altering the electric field and introducing conductive droplets and counter electrodes [165]. Du et al. [166] fabricated electro-responsive CMs-P membranes by coating carbon nanofiber membranes with poly(3-methylthiophene) (P(3-MTH)). The prepared membranes were reversibly doped and de-doped with P (3-MTH) by ClO4^−^ under electrical stimulation to realize the mutual transition of hydrophilicity and dehydration of the membranes (Fig. 10a). Light stimuli-responsive membranes can be irradiated with ultraviolet light (UV) or visible light (Vis) to alter the surface wettability of the membrane. Employing light as an external stimulus offers excellent selectivity, rapid reaction times, and controllability, making photostimulable responsive membranes a cost-effective and easily operated option with high stability [167–169]. Chen and his co-workers proposed the grafting of azobenzene groups on silica (SiO_2_) roughened polypropylene (PP) membranes (SiO_2_ NPs-grafted PP). By combining SiO_2_ NPs-grafted PP membranes and photosensitive 7-[(trifluoromethoxyphenylazo)-phenoxy]-pentanoic acid (CF_3_AZO), PP-g-SiO_2_ NPs/CF_3_AZO membranes that are also photoresponsive were prepared [170]. PP-g-SiO_2_ NPs/CF_3_AZO membranes can be alternatively irradiated with UV and visible light, resulting in the conversion of cis and trans states of CF_3_AZO and accomplishing alternate hydrophilic and hydrophobic interchanges on the surface of the membrane (Fig. 10b). Ion stimuli-responsive membranes are commonly synthesized using polymers carrying charged groups that can switch between hydrophilic and hydrophobic by cation/anion exchange or by adding ions to the membrane surface [128, 171]. Poly(ionic liquid)s (PILs) are used as the main ion-stimulating responsive polymers due to their favorable characteristics, including good thermal stability, solubility, catalytic activity, and non-flammability [172, 173]. Gao et al. prepared the hydrophilic poly(1-vinyl-3butylimidazolium acrylate)-based membrane (PILM-1) and the hydrophobic poly(1-vinyl-3octylimidazolium hexafluorophosphate)-based membrane (PILM-5) by controlling the length of the alkyl chains of cations and anions of the PILs using a one-step photopolymerization method (Fig. 10c). Both membranes exhibited excellent separation efficiencies. They could be assembled to achieve continuous oil–water separation [174].

## Conclusion

Membrane separation technology, recognized for its simplicity, cost-effectiveness, and efficiency, is considered one of the most effective means of separating oil and water. This article presents fundamental design concepts and the research advancements in oil–water separation membranes, including high-flux, high-efficiency, fouling-resistant and stimuli-responsive oil–water separation membranes. Despite significant progress in the research on oil–water separation membranes, challenges remain.

1. The critical problem is membrane fouling. Oil is highly susceptible to adsorption on the membrane, leading to pore clogging, which rapidly decreases flux and separation efficiency. Therefore, the antifouling ability of the membrane is of utmost importance. Improving the long-term anti-adhesive contamination ability of the membrane surface to oil and surfactant in the continuous separation process, and realizing the practical application of the oil–water separation membrane is among the challenges for future research.
2. The field requires scalable processes for membrane fabrication such as interfacial polymerization(IP) [175]. In order to make membrane fabrication processes such as the IP process more scalable not only in the field of liquid separation but also in other fields, several approaches can be considered. One of these approaches is the choice of materials, which involves identifying compatible monomers and substrates for the desired application. To explore the versatility, monomers with different chemical functions can be selected to meet the requirements of various fields. The process should be optimized, and the process parameters, such as temperature, pressure, and reaction time, should be tailored to the specific requirements of different applications to achieve the desired material properties and performance characteristics. In addition, the process can be used to achieve membrane surface modification and functionalization to fabricate tailor-made coatings with specific properties (such as hydrophobicity, biocompatibility or antimicrobial activity) for applications in different fields.
3. Further in-depth investigation into the mechanism of oil–water separation is required. Currently, most research is focused on membrane design and preparation, with little understanding of how oil droplets are demulsified and coalesced on the membrane surface during oil–water separation. Exploring the mechanism of oil–water separation can provide a theoretical direction for designing high-performance membranes, thus breaking through the application bottleneck of oil–water separation membranes in the future. Liquid phase transmission electron microscopy can provide new insights into membrane fouling and oil–water separation mechanism at the nanoscale, which may address the aforementioned challenges from a micro perspective. This paper aims to increase researchers' interest in this field and aid in the future research and development of oil–water separation membranes.

## Data Availability

Data can be obtained from authors under reasonable request.
